# Role of nursing professionals for parenting development in early childhood: a systematic review of scope

**DOI:** 10.1590/1518-8345.3031.3213

**Published:** 2019-12-05

**Authors:** Kesley de Oliveira Reticena, Vânia do Nascimento Tolentino Yabuchi, Maria Fernanda Pereira Gomes, Lucíola D’Emery Siqueira, Flávia Corrêa Porto de Abreu, Lislaine Aparecida Fracolli

**Affiliations:** 1 Universidade de São Paulo, Escola de Enfermagem, São Paulo, SP, Brazil.; 2 Universidade Paulista, UNIP Assis, Assis, SP, Brazil.; 3 Universidade Federal de São Paulo, Escola Paulista de Enfermagem, São Paulo, SP, Brazil.; 4 Universidade Paulista, UNIP Limeira, Limeira, SP, Brazil.

**Keywords:** Nurse Practitioners, Nursing Role, Child Care, Child Development, Nursing Practice, Parenting, Profissionais de Enfermagem, Papel do Profissional de Enfermagem, Cuidados Infantis, Desenvolvimento Infantil, Prática de Enfermagem, Parentalidade, Enfermeras Practicantes, Rol de la Enfermera, Cuidado de los Niños, Desarrollo Infantil, Práctica de Enfermería, Responsabilidad Parental

## Abstract

**Objective:**

map available evidence regarding the role of nursing professionals in early childhood care through the development of parenting.

**Method:**

systematic review of scope, with selection of studies about the role of nursing professionals for the development of parenting in the context of early childhood care, using a standardized data extraction tool and qualitative thematic analysis.

**Results:**

nineteen studies were included, showing the role of nursing professionals covers nine dimensions: promoter of parental role construction; guidance and support for the implementation of physical health care; guidance for promoting safe environment; application of theories, principles and methods of maternal and child programs; development of therapeutic relationships; implementation of maternal and child care management; promotion of access to support network; guidance for the life course of parental figures; and use of scientific evidence to guide practice.

**Conclusion:**

nursing professionals offer important knowledge and significant practices for the development of parenting in early childhood care. These findings provide the basis for improving the clinical practice of these professionals, showing relevant areas of action and interventions to early childhood.

## Abstract (pt)

**Objetivo:**

mapear a evidência disponível sobre a atuação do profissional de enfermagem no cuidado à primeira infância por meio do desenvolvimento da parentalidade.

**Método:**

revisão sistemática de escopo, com seleção de estudos sobre a atuação de profissionais de enfermagem contemplando o desenvolvimento da parentalidade, no contexto de cuidados à primeira infância. Utilizou-se ferramenta padronizada de extração de dados e análise temática qualitativa.

**Resultados:**

foram incluídos 19 estudos que mostraram que a atuação de profissionais de enfermagem perpassa por nove dimensões: promotor da construção do papel parental; orientações e apoio para implementação de cuidados de saúde físicos; orientações para promoção de ambiente seguro; aplicação de teorias, princípios e métodos dos programas materno-infantis; estabelecimento de relações terapêuticas; implementação da gestão da atenção materno-infantil; promoção de acesso à rede de apoio; orientação para o curso da vida das figuras parentais; e uso de evidências científicas para orientar a prática.

**Conclusão:**

a atuação de profissionais de enfermagem abrange importantes saberes e significativas práticas para o desenvolvimento da parentalidade no cuidado à primeira infância. Os achados possibilitam embasamento para melhoria da prática clínica desses profissionais, ao mostrarem as áreas de atuação e intervenções relevantes para a primeira infância.

## Abstract (es)

**Objetivo:**

localizar la evidencia disponible sobre la actuación del profesional de enfermería en el cuidado de la primera infancia mediante el desarrollo de la parentalidad.

**Método:**

revisión sistemática de alcance, con selección de estudios sobre la actuación de los profesionales de enfermería en el desarrollo de la parentalidad durante el cuidado de la primera infancia. Se utilizó una herramienta de extracción de datos estandarizada y un análisis temático cualitativo.

**Resultados:**

se incluyeron 19 estudios que mostraron que en la actuación de los profesionales de enfermería atraviesan nueve dimensiones: promotora de la construcción del rol parental; orientaciones y apoyo a la implementación de cuidados de salud físicos; orientaciones para promover un ambiente seguro; aplicación de teorías, principios y métodos de programas maternoinfantiles; establecimiento de relaciones terapéuticas; implementación de la gestión de la asistencia maternoinfantil; fomento al acceso a la red de apoyo; orientación para el curso de vida de las figuras parentales; y uso de evidencias científicas para orientar la práctica.

**Conclusión:**

la actuación de los profesionales de enfermería abarca conocimientos importantes y prácticas significativas para el desarrollo de la parentalidad en el cuidado de la primera infancia. Los hallazgos proporcionan un soporte para mejorar la práctica clínica de estos profesionales mostrando las áreas de actuación e intervenciones relevantes para la primera infancia.

## Introduction

Parental care during childhood can affect a child’s development and life, with possible impact on health and economic and social consequences due to influences in the early period of human development^(^ 1 ^)^.

Individual and social circumstances experienced by vulnerable families can be a challenge to a successful start for children and disrupt long-term socioeconomic stability^(^ 2 ^)^. Besides, studies show positive correlations between poor family structure and development of emotional, behavioral and cognitive problems in children^(^ 3 ^)^.

Parenting is described as activities aiming to promote the survival and full development of children performed by their reference adults^(^ 4 ^)^, who are responsible for caring, stimulating, providing information, loving, imposing limits, ensure autonomy and preparing the child for the challenges and opportunities of present and adult life^(^ 4 ^)^.

Interventions during pregnancy and childhood to help reference adults acquire positive parenting skills and create safe and healthy environments are the goals of several nurse home visiting programs^(^ 5 ^-^ 6 ^)^.

These nurse home visits promote strong connections with visited families, so nursing professionals become reliable for them^(^ 7 ^)^, with the possibility to exchange knowledge from different fields, establish effective communication channels with various social sectors and promote effective health care strategies.

Nursing is recognized for its ability to understand and take care of human beings in general, assisting them in their health issues^(^ 8 ^)^. However, according to a primary study found in the literature, nursing professionals are not prepared to meet the demands of their role in child health care^(^ 9 ^)^.

Despite presenting validated instruments for the diagnosis of support to parental figures and target of nursing interventions^(^ 10 ^)^, the literature is not clear about the role of nursing professionals for parenting development in early childhood, which justifies the development of this study, considering the relevance of parenting development and the role played by nursing professionals in supporting parental figures in this process of early childhood care.

In addition, based on international experience, maternal and child health programs focused on building positive parenting skills have more successful results when developed by nursing professionals^(^ 11 ^-^ 13 ^)^.

A search conducted in the systematic review database of the Joanna Briggs Institute, the Cochrane Library and the online medical literature search and analysis system (PubMed/MEDLINE) in November 2017 did not identify any systematic review or published review protocols related to this topic. In case of absence of clear evidence of the role of nursing professionals in parenting development in early childhood care, these professionals miss the opportunity to intervene based on evidence in child development. Therefore, a scope review is needed to map studies on this topic.

Then, the following review question was developed: What is the nursing professional role in parenting development in early childhood care? Therefore, the objective of this scope review was to map available evidence about the role of nursing professionals in early childhood care through parenting development.

## Method

This review used the P-C-C strategy to formulate the review question, as proposed by the Joanna Briggs Institute, where “P” refers to the population/participants, “C” is the concept to be investigated, and “C” refers to the context. Therefore, this review considered the studies conducted with the population of nursing professionals that addressed their performance in early childhood care through parenting development.

For the purposes of this study, the source of information included existing literature, such as published or unpublished quantitative or qualitative primary research studies, systematic reviews, and research reports. Information in Portuguese, English and Spanish were considered for inclusion. No date periods were applied.

The research strategy and the whole development process of this study adopted the systematic review methodology proposed by the Joanna Briggs Institute. So a three-step research strategy was used. First, an initial search was performed on PubMed/MEDLINE only to identify articles about this topic, followed by an analysis of the words contained in the titles and abstracts and the index terms used to describe these articles. This procedure supported the development of a search strategy, including key words and index terms.

Descriptors and key words used in the search strategies, with Boolean connectors AND and OR, were: Nurse Practitioners, Nurses, Nurse’s Role, Professional Competence, Parenting, Child Care, Children Care, and Child development, adapted to each research source. The search strategies are detailed in documents maintained by the authors and can be disclosed, if necessary. The databases and sources analyzed in this review were: the Joanna Briggs Institute’s systematic review database, Cochrane’s systematic review database, PubMed/MEDLINE, Cumulative Index to Nursing and Allied Health Literature (CINAHL), and Virtual Health Library (VHL). Search for unpublished studies included Google scholar and different dissertation and thesis databases. 

Then the second step was conducted, using the key words and index terms in the search. In the third step, the reference list of all selected texts was traced for further studies. 

Data were extracted from documents included in the scope review by three independent reviewers using a standardized data extraction tool adapted from the tool proposed by the Joanna Briggs Institute. Data extracted included specific details about population, concept, context, methods, and significant results for the scope analysis question. Any disagreement among the reviewers was resolved through critical discussion among them. The authors of studies were contacted for missing or additional data when needed. The preliminary data extraction tool was modified and revised as necessary during the data extraction process for each study included in this review. When the results of the same study were reported in more than one article, only one was included. The study selection process and the last search were performed in June and July 2018.

Extracted data are presented in figures and include date of publication, title, study design, and country. A qualitative thematic analysis was performed to provide an overview of the literature, and is presented in a figure with results describing the dimensions of nursing professional role. The results are discussed and related to practice and research.

This review did not involve human beings, so it was not submitted to the research ethics committee. In addition, for being a systematic scope review, it did not require a methodological quality assessment of included studies. This study observed the Standards for Quality Improvement Reporting Excellence 2.0 (SQUIRE 2.0) and was reviewed according to the systematic review checklist - Preferred Reporting Items for Systematic Reviews and Meta-Analyses (PRISMA)^(^ 14 ^)^.

## Results

In total, 477 studies were identified in databases and 10 additional records were obtained from other sources. After excluding repeated articles, titles of 299 documents were read to check them against the inclusion criteria. Of these, 188 studies were selected for abstract reading and later 49 were fully read. Of these, 30 were excluded because they did not meet the inclusion criteria and the objectives, so 19 were included in this review.

**Figure 1:**
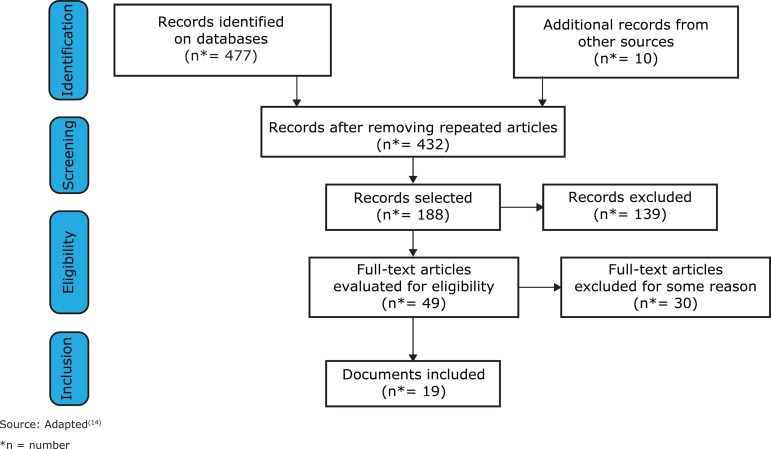
PRISMA flow diagram illustrating the four different phases of study selection and inclusion process for the systematic review

Source: Adapted^(^14^)^ *n = number

Figure 1 shows the document selection process in a PRISMA flow diagram^(^ 14 ^)^:

The studies included in this review^(^ 15 ^-^ 33 ^)^ were produced from 1998 to 2016, most of them^(^ 14 ^)^ in the last 10 years. Seven studies were conducted in Portugal, five in the United States, two in Australia, one in England, one in Sweden, one in Scotland, one in Canada and one in Jamaica, Antigua and Saint Lucia. Figure 2 details the characteristics of these studies:

**Figure 2:** Documents included in this review showing their year of publication, title, design, and country. São Paulo, SP, Brazil, 2018

Year	Title	Study design	Country
1998^(^ 15 ^)^	Reversing Growth Deficiency in Children: The Effect of a Community-based Intervention	Randomized controlled trial	United States
1999^(^ 16 ^)^	Prenatal and Infancy Home Visitation by Nurses: Recent Findings	Randomized trial	United States
2000^(^ 17 ^)^	Assessing the Impact of Pediatric-Based Developmental Services on Infants, Families, and Clinicians: Challenges to Evaluating the Healthy Steps Program	Evaluation research	United States
2000^(^ 18 ^)^	Does home visiting improve parenting and the quality of the home environment? A systematic review and meta analysis	Systematic review	England
2000^(^ 19 ^)^	Supportive and non supportive qualities of child health nurses’ contacts with strained infant mothers	Quantitative qualitative research	Sweden
2009^(^ 20 ^)^	Toward Better Beginnings Enhancing Healthy Child Development and Parent–Child Relationships in a High-Risk Population	Intervention study and comparison	United States
2010^(^ 21 ^)^	Parentalidade Positiva e Enfermagem: Revisão Sistemática da literature (Positive Parenting and Nursing: Systematic literature Review)	Systematic review	Portugal
2010^(^ 22 ^)^	The role of child health nurses in supporting parents of young infants	Quantitative evaluation research	Australia
2012^(^ 23 ^)^	Apoiar na parentalidade positiva: áreas de intervenção de enfermagem (Support in positive parenting: areas of nursing intervention)	Correlational study	Portugal
2013^(^ 24 ^)^	Satisfação dos Pais sobre a promoção da Parentalidade realizada pelo Enfermeiro da Famíli (Parent satisfaction on parenting promotion by family nurse)	Cross-sectional descriptive study	Portugal
2013^(^ 25 ^)^	A transição no exercício da parentalidade durante o primeiro ano de vida da criança: uma teoria explicativa de enfermagem (Transition in parenting practice during the child’s first year of life: an explanatory theory of nursing)	Qualitative study	Portugal
2014^(^ 26 ^)^	Implementation and Randomized Controlled Trial Evaluation of Universal Postnatal Nurse Home Visiting	Randomized trial	United States
2015^(^ 27 ^)^	Integrating a Parenting Intervention With Routine Primary Health Care: A Cluster Randomized Trial	Randomized trial	Jamaica, Antigua and Saint Lucia
2015^(^ 28 ^)^	Supporting Pakistani and Chinese families with young children: perspectives of mothers and health visitors	Qualitative research	Scotland
2015^(^ 29 ^)^	Enfermeiro de Saúde Familiar e a comunicação: Transição para a Parentalidade (Family nurse and communication: transition to parenting)	Descriptive report	Portugal
2015^(^ 30 ^)^	Intervenção dos enfermeiros na capacitação parental no relacionamento pais-filhos (Nurse’ intervention in parent training in parent-child relationship)	Systematic review	Portugal
2016^(^ 31 ^)^	Improving children’s health and development in British Columbia through nurse home visiting: a randomized controlled trial protocol	Randomized controlled trial	Canada
2016^(^ 32 ^)^	An interprofessional exploration of nursing and social work roles when working jointly with families	Exploratory case study	Australia
2016^(^ 33 ^)^	Das competências parentais à promoção do desenvolvimento infantil na primeira infância: desafios para o Enfermeiro Especialista (From parenting skills to promoting early childhood development: challenges for specialist nurses)	Descriptive report	Portugal

All documents included in this review address the context of early childhood care. Figure 3 shows the role of nursing professionals for the development of parenting, described in nine dimensions:

**Figure 3:** Roles of nursing professionals for parenting development and their dimensions. São Paulo, SP, Brazil, 2018

**DIMENSION 1** ** ** **Promoter of parental role construction**	- Conduct parental education by promoting knowledge to parental figures about the child’s growth and development, their motor, personal-social and language skills, videos and videotaping may be used as strategies. - Guide to stimulate children through touching, speaking and playing. - Identify temperament traits and how to manage them with reaffirmation of authority (apply rules, routine, rewards). - Promote parent-child interaction, facilitating parental figure understanding of children’s communication signals to promote emotional and cognitive development. - Enable increased satisfaction in parenting. - Enable reduction of maternal depression and stress related to parenting. - Provide support to positive parenting role, with guidance to improve parenting skills. - Provide early guidance on role transition. - Promote effective teaching of behavioral parent skills for easier incorporation of the role. - Assist parental figures in proper parenting management. - Encourage parents in their role of raising children. - Help parents solve problems, perform parent tasks and understand situations in the perspective of development. - Plan interventions in partnership with parents to strengthen marital and parental relationship. - Encourage parent reading for children for cognitive and language development. - Guide mothers about behaviors that promote family bonding, development and proper growth without disrespecting culture. - Positively reinforce all efforts made and gains achieved in the harmonious development of the child and healthy performance of the parental role. - Evaluate conflictual dimensions related to the role. - Plan family rituals. - Promote adaptive strategies – coping in the family. - Be aware of the dyad relationship between parental figures and children and possible behavioral problems. - Reduce behavioral problems in children, improve relationships between parental figures and children and prevent future problems. - Improve detection, support and referral of families and children with behavioral problems in their early stages. - Involve parental figures in decision-making and care process. - Identify and understand the main challenges and needs of parental figures in the exercise of parenting.
**DIMENSION 2** ** ** Guidance and support for the implementation of physical health care	- Address concerns of mothers and family members about complications of pregnancy, labor and delivery, and physical health of babies. - Stimulate healthy growth and development of children. - Teach mothers and family members how to identify signs of health problems and clinical signs (temperature), and seek health centers in case of changes. - Perform early detection and treatment of disorders. - Evaluate women’s smoking habits and consumption of alcohol and illicit drugs, facilitating reduction of such use through behavioral strategies. - Teach women how to identify signs and symptoms of pregnancy complications, encourage women to inform health team about these conditions, and facilitate treatment completion. - Pay attention to urinary tract infections, sexually transmitted infections, hypertensive disorders (conditions associated with poor birth conditions). - Help mothers and other caregivers improve physical and emotional care to their children. - Promote better preventive maternal care. - Promote the dissemination of information/resources about physical health and child development. - Provide care related to key areas for the promotion of child development. - Provide care that combines health promotion, disease prevention and clinical responsibility and actions targeting family members. - Analyze the presence of risk factors in the family. - Promote educational interventions in specific risk areas. - Provide health education about different topics: breastfeeding, feeding, prevention of infectious diseases, prevention of accidents, recreational and leisure activities, hygiene and comfort care, bowel elimination pattern. - Emphasize the importance of observing the national immunization schedule and regular child/youth health visits. - Conduct nutritional assessment and provide guidance. - Guide postpartum mothers about newborn feeding and care.
**DIMENSION 3** ** ** Guidance for promoting safe environment	- Conduct environmental education (housing hygiene and health promoting environment). - Provide guidance for the implementation of safe family environment (smoke detector, tap water temperature, protection on windows and stairs, safe place for children to sleep). - Instruct and encourage the family for home management (definition of monthly resources for food, transportation and items of basic needs). - Provide guidance to promote a place with an area where children can play, for their meals, promoting a daily routine. - Plan interventions in partnership with parents, creating an environment that contributes to development and well-being.
**DIMENSION 4** ** ** Application of theories, principles and methods of maternal and child programs	- Conduct home visits. - Use protocols of family supervision programs. - Perform evaluation in a room for children exam. - Provide telephone support to answer questions. - Promote discussion groups with parental figures for specific themes. - Distribute explanatory material. - Visit the hospital during pregnancy and after the birth. - Supervise child development in a structured program of child monitoring in all stages of child development. - Apply program guidelines to visits. - Divide home visit time to cover all domains defined by the program. - Apply the theoretical framework that supports the program. - Support a specific number of cases, as defined in the program.
**DIMENSION 5** ** ** Development of therapeutic relationships	- Develop friendly and trustful relationships with mothers and other family members. - Implement a therapeutic relationship with pregnant women to deal with interpersonal situations and problem resolution. - Offer emotional support, interest and attention to the mother/father and their baby. - Provide informational support about baby care. - Respect the decisions of parental figures. - Enhance self-esteem and independence of parental figures allowing them to take care of their children. - Provide nursing care that implies an interrelation with the child and family. - Promote expressive communication of emotions. - Learn about the needs and desires of parental figures.
**DIMENSION 6** ** ** Implementation of maternal and child care management	- Manage complex clinical situations. - Manage and organize resources for maximum autonomy of those targeted by the intervention. - Assume the role of manager (of health/disease processes, of community resources), educator, emotional support, enabling family empowerment (at intrapersonal, interpersonal and organizational levels), with an intervention from the microsystem level to the macrosystem level. - Act in challenging and complex personal and social situations of families.
**DIMENSION 7** ** ** Promotion of access to support network	- Help women build supportive relationships with family members and friends. - Link women and their families with other services. - Provide information about community resources that parental figures can use in child care. - Promote a connection with community services and resources. - Provide the family with guidance about social services. - Perform interprofessional actions with social workers.
**DIMENSION 8** ** ** Guidance for the life course of parental figures	- Help women define their goals and solve problems that may affect the continuity of their education, job search and planning of future pregnancy. - Help women improve behavior related to health, care and life course development. - Promote building of social capital. - Promote household income management. - Promote improvements in the behavior of women and family members that affect the life course of parental figures.
**DIMENSION 9** ** ** Use of scientific evidence to guide practice	- Use scientific methodology. - Use a child- and family-centered conceptual model. - Interact with families through an organized, dynamic and systematic method of critical thinking about family health. - Collect data about every family that allow problem identification and the formulation of nursing diagnoses. - Assume a comprehensive character, integrating perspectives and actions of technical and scientific nature that are specific to this period of development and that can fulfill emotional and social needs. - Have systemic models of family guidance that recognize the interdependence of processes affecting the family ability to ensure health development of family members. - Have deep and specialized knowledge about health, child development and methodologies allowing positive and interactive relationships with the parental figures.

## Discussion

This systematic review of scope involved the implementation of three steps: an initial investigation to identify articles about the topic and assist in the development of a search strategy, search on all databases using key words and index terms, and finally tracking of all selected studies, totaling 19 documents.

For the development of parenting in early childhood care, the role of nursing professionals covers nine dimensions: (1) promoter of parental role construction; (2) guidance and support for the implementation of physical health care; (3) guidance for promoting safe environment; (4) application of theories, principles and methods of maternal and child programs; (5) development of therapeutic relationships; (6) implementation of maternal and child care management; (7) promotion of access to support network; (8) guidance for the life course of parental figures; and (9) use of scientific evidence to guide practice.

Several health care initiatives aim to promote areas with an impact on child development. In this sense, the dimensions of the role of nursing professionals presented in this study allow targeted interventions for the development of parenting and child health promotion, in agreement with the objectives of existing initiatives in this field.

Regarding the dimension of promoter of parental role construction, positive parenting covers several attributions of reference adults in child care, which are critical for child health and development. In the first years of life, a child’s brain presents high learning potential^(^ 10 ^,^ 34 ^)^, which is an opportunity for parental figures to optimize child development^(^ 10 ^)^; however, in some cases, they need support and guidance to play their role properly^(^ 34 ^)^. In the meantime, nursing professionals can provide support, articulated with other professionals and social sectors, helping and preparing parental figures to perform their duties.

Guidance and support for the implementation of physical health care and guidance for promoting safe environment were also addressed as roles of nursing professionals in the explored context. Such attributions can generate better results through strategies that facilitate their implementation, such as home visits. A study shows home visits by nursing professionals during the prenatal period and first years of the child help reduce all-cause mortality among mothers and avoidable mortality among children^(^ 35 ^)^.

Besides home visits, other strategies are used in the application of theories, principles and methods of maternal and child programs, such as telephone support, discussion groups and use of videos. Then, actions should be developed by nursing professionals to achieve the goals of specific programs. The literature demonstrates the role of nursing professionals should be planned in detail before its practical implementation^(^ 36 ^)^.

The dimensions of the role of nursing professionals parenting development are closely connected. For example, with the development of therapeutic relationships, guidance for the life course of parental figures and action in other care dimensions are facilitated. In this context, initiatives focusing on early childhood through parenting development have had long-term effects on prenatal health, child health and development, and the mother’s life course^(^ 34 ^)^.

However, nursing professionals often act in the dimensions of implementing the role of manager and promoting access to support network, especially with more vulnerable families in complex situations. Nursing professionals, due to their ability to address different areas and services, tend to assume the role of a ‘case manager,’ helping parental figures access and use other community services^(^ 7 ^)^.

To explain all these dimensions of action, scientific evidence is used to guide their practice, as professionals need support to develop and maintain their skills and a knowledge base for high-quality evidence-based practice^(^ 9 ^)^ in order to perform their role effectively.

Ideally, early childhood development services should be offered holistically and integrally across all relevant sectors, without fragmentation, to enable young children to thrive^(^ 34 ^,^ 37 ^)^. Policies to reduce poverty and strengthen family resources can create a favorable environment to promote, protect and support early childhood development^(^ 38 ^)^.

The cost of inaction is high in terms of early childhood development, considering that insufficient investment in the development of physical, cognitive, emotional and social capacities of children has implications not only for them but for society in general, enhancing inequalities and social divisions^(^ 39 ^)^. Therefore, nursing professionals, articulated with other professionals and social sectors, are key elements in this issue, as they have different care abilities to offer children and families.

In this sense, this systematic scope review significantly contributes to scientific development of nursing by demonstrating implications for nursing practice and research, enabling better support and improved care to families, and showing the areas of practice and interventions performed. In addition, it offers definitions of phenomena that can be incorporated into the Nursing Care Systematization (SAE - *Sistematização da Assistência de Enfermagem*).

A gap was identified regarding the definition of abilities of nursing professionals in the field analyzed, since the reviewed studies address how these professionals acts, and not how their performance should be, which is a limitation of this study. Then, future studies could use these findings to develop nursing professional skills for early childhood care through the development of parenting.

## Conclusion

This systematic scope review mapped the evidence available about the role of nursing professionals in early childhood care through the development of parenting, identifying nine different dimensions of this role. This review found nursing professionals can contribute to the development of parenting in early childhood care, given the relevance and amplitude of their role.

Parenting development requires different areas of influence and family dimensions and society segments, with nursing professionals holding a privileged position of contact with the child and family members at different levels of care.

By showing the areas of practice and interventions performed by nursing professionals, with the same objective, in different regions of the world, this study provides a better understanding and improvements in clinical practice and SAE implementation. It supports the development of research to build skills of nursing professionals in early childhood care, through the development of parenting, given the gap identified in this area.
